# The effect of a mobile-learning curriculum on improving compliance to quality management guidelines for HIV rapid testing services in rural primary healthcare clinics, KwaZulu-Natal, South Africa: a quasi-experimental study

**DOI:** 10.1186/s12913-022-07978-4

**Published:** 2022-05-09

**Authors:** Nkosinothando Chamane, Ropo Ebenezer Ogunsakin, Tivani Phosa Mashamba-Thompson

**Affiliations:** 1 Department of Public Health Medicine, School of Nursing and Public Health, University of KwaZulu-Natal, Durban, South Africa; 2 Faculty of Health Sciences, Gauteng Province, University of Pretoria, Prinshof Campus, Pretoria, South Africa

**Keywords:** Mobile Learning, Quality rapid HIV testing and Quasi-experimental design

## Abstract

**Background:**

Despite significant achievements made towards HIV testing, linkage to antiretroviral therapy treatment and viral load suppression, the Sub-Saharan region of Africa continues to be reported to have the highest prevalence of HIV/AIDS, with over 26 million people living with the disease. In light of the added burden on already overwhelmed health systems due to the Covid-19 pandemic, maintaining the reliability and accuracy of point-of-care diagnostics (POC) results is crucial to ensure the sustainability of quality service delivery. The integration of technology-based interventions into nurse education curricula is growing, to help prepare students for the current practice environment which requires access to large amounts of information. The aim of this study was to determine the effect of a Mobile Learning (mLearning) Curriculum on improving the quality of HIV rapid testing services in rural clinics of KwaZulu-Natal (KZN), South Africa.

**Methods:**

To achieve the aim of this study, pre-test and post-test audits were conducted in a quasi-experimental design. Eleven clinics of KZN, with the highest availability and usage of POC diagnostics were selected from a cross-sectional study survey to constitute the sample of this study. The World Health Organization On-site Monitoring Checklist-Assessment of Quality System was adapted and used as an audit tool to evaluate four key quality components. The effect of the mLearning curriculum on HIV testing quality improvement was determined through statistically comparing pre-audit and post-audit results. The independent samples t-test and the Levene’s test were employed to evaluate the equality of measured variables for the two groups. The relationships between variables were estimated using the Pearson pair wise correlation coefficient (p) and correlations were reported as significant at *p* < 0.05.

**Results:**

A total of 11 clinics was audited at the pretest and 7 clinics were audited post-piloting of the mLearning curriculum. The estimated level of compliance of the participating clinics to quality HIV rapid testing guidelines ranged between poor and moderate quality. The mLearning curriculum was shown to have no statistically significant effect on the quality of POC diagnostic services provided in rural clinics of KZN.

**Conclusion:**

The mLearning curriculum was shown to have no statistically significant effect on the quality of HIV rapid testing services provided in participating clinics; however, multiple barriers to the full adoption of the piloted curriculum were identified. The provision of reliable technology devices and improved internet connection were recommended to enhance the adoption of technology-based interventions necessary to improve access to relevant learning material and updated information.

**Supplementary Information:**

The online version contains supplementary material available at 10.1186/s12913-022-07978-4.

## Background

The sub-Saharan Africa region continues to be reported to have the highest prevalence of HIV/AIDS globally, having over 26 million people living with HIV/AIDS in 2019 [1]. However, a lot has been achieved in the field of HIV testing and disease management over the years. In 2019, an average of 81% of people living with HIV knew their HIV status, amongst these, 82% were accessing antiretroviral treatment and among those accessing treatment, 88% were reported as virally suppressed [2]. Rapid HIV testing has also been shown to be associated with decreased mother-to-child transmission of HIV and the increased linkage to Anti-retroviral treatment (ART) both locally and globally [3, 4]. Currently the advent of the Covid-19 pandemic has been reported to add an extra burden on overwhelmed health systems, posing a risk of severe disruptions to HIV services in sub-Saharan Africa [5, 6]. This is assumed to significantly increase mortality rates due to interruptions of ART supply [5].

In light of the significant role played by HIV rapid tests in improving access to HIV care and the foreseen challenges in resource-limited settings due to the Covid-19 pandemic, maintaining reliability and the accuracy of test results to ensure sustainability of quality service delivery remains crucial. The World Health Organisation (WHO) guidelines for assuring the accuracy and reliability of HIV rapid testing services highlight organisation, personnel, quality control and assessment amongst critical components necessary to assure quality service provision in all HIV testing facilities [7]. Poor adherence to guidelines has been observed to highly compromise the quality of HIV testing services provided in resource- limited settings of South Africa [8]. In the previous study, relevant stakeholders with experience in HIV testing and management also highlighted challenges to adherence to quality requirements due to lack of opportunities of continuous professional development (CPD) [9].

The South African National Department of Health has conducted workshops periodically to train and develop primary healthcare (PHC) clinic workers on HIV testing. They have also distributed relevant policy documents on HIV testing and management [10]. However, adherence to quality management guidelines in clinics situated in deep rural areas remains a challenge. The aim of this study was to determine the effect of a Mobile Learning (mLearning) Curriculum on improving quality components of HIV rapid testing services in rural clinics of KwaZulu-Natal (KZN), South Africa. It is anticipated that the findings of this study will inform policy makers and curriculum designers in the designing and implementation of an evidence-based curriculum to improve the quality of POC diagnostic services in resource-limited settings and hence contribute towards strengthening of health systems.

## Materials and methods

### Study design

In this study pre-test and post-test audits were conducted in a quasi-experimental design to evaluate the effectiveness of an mLearning curriculum on the improvement of quality components (organization, personnel, process improvement,and service and satisfaction) of HIV testing services in representative rural clinics of KZN. Quasi-experimental designs are defined as forms of experimental research used to establish a cause and/or effect of an intervention on a population without randomisation [11]. This design was selected as appropriate to achieve the aim of the study because it has been reported to add substantial value to health research and evidence synthesis. Furthermore, this design has been shown to be effective in determining the effects of interventions in ‘real life’ [12].

### Sampling strategy

This study was conducted as a follow up on a cross-sectional study, which involved an audit of 100 rural PHC clinics in rural KZN [13]. The cross-sectional study was aimed at identifying barriers and challenges related to the implementation of POC diagnostics in the province. Eleven clinics (one clinic per KZN district) that have the highest availability and usage of POC diagnostics were selected from the survey and these constituted the sample of this study.

### Intervention

The mLearning curriculum was designed with the intention to provide accessible and updated quality HIV POC diagnostics training to PHC nurses and HIV lay councillors in the rural clinics of KZN. The mLearning curriculum content consisted of three learning units (Counselling, HIV testing process and quality requirements), activities, an online quiz and an online survey.To deliver this curriculum content, an online education platform (Learning Environment) “Moodle” was utilised to create an interactive online course for participants to experience and give feedback on the content provided. Moodle is a free and open-source learning management system written in PHP and distributed under the GNU General Public License [14, 15]. This platform provides for various forms of electronic learning (eLearning), including mLearning through the Moodle app and web browser.

For ethical purposes, dummy usernames and passwords were created and loaded onto Moodle and sent to participants for access to the online course. Real email addresses were submitted to have accounts activated but participants’ personal identities were not loaded onto the system. Through contact sessions, participants were orientated on accessing the course via the Moodle app and the standard route for distance learning. The course was open for a period of four months, which included an additional extension of one month to accommodate participants reported to be experiencing connection problems.

Learning Outcomes: After the course the participants would be able to:

- Define terms, including: HIV infection, AIDS, antibody, antigens, rapid test, window period.
- Explain the need for quality HIV testing in the prevention and treatment of HIV/AIDS.
- Identify and prevent factors that may compromise the quality of HIV rapid testing.
- Understand the responsibilities of the health worker in the prevention and detection of errors prior to, during and post testing.
- Propose strategies to improve the training program.

More detail on the design and piloting of this intervention has been published in our recent article [16].

### Data collection procedure

**Fig. 1:**
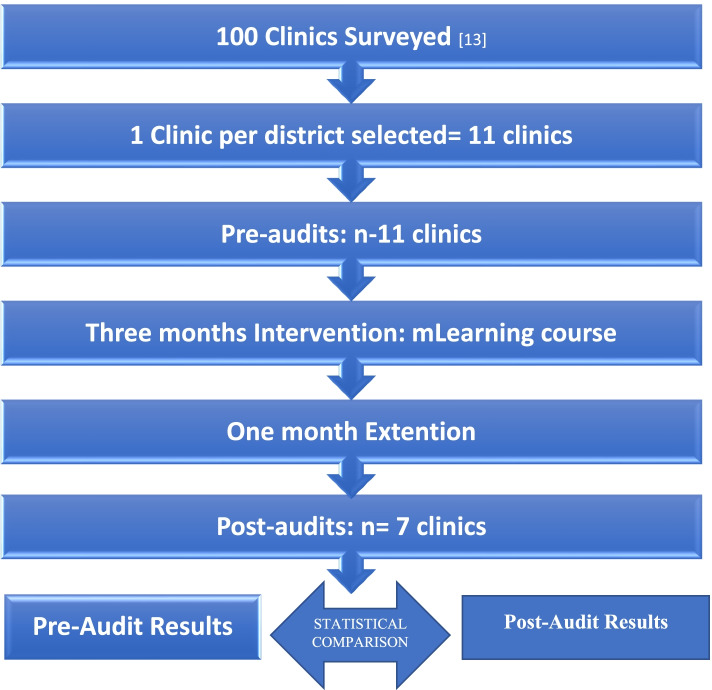
Study process flow chart

The effect of the mLearning curriculum was evaluated by comparing pre-audit and post-audit results using a pre-test/post-test design. Pre-test and post-test data were collected using an audit tool as described below. Pre-test data was generated prior to participating in the three months mLearning course. Post-test data was generated after four months of open access to the mLearning course. The audit team consisted of the study primary investigator (PI), a trained research assistant, professional nurses, HIV lay counsellors (where available) and clinic managers. The audit team worked together to conduct audits. The PI and research assistant presented and explained the components of the checklist. The clinic managers or their representatives and/or HIV lay counsellors responded to the questions on the checklist and went on to show the PI and research assistant different items as requested on the audit checklist. To ensure efficiency, relevant clinic personnel were informed of the purpose of the audits and the procedure to be followed prior to clinic visits. The flowchart of our study process is shown in Fig. 1 below:

### Audit tool and scoring guide

The World Health Organisation (WHO) provides guidelines for assuring the accuracy and reliability of HIV rapid testing services in rural and resource-limited settings [17]. Furthermore the South African Department of Health has adopted these guidelines to frame national policies towards ensuring provision of quality POC diagnostic services [18]. In this study the On-site Monitoring Checklist-Assessment of Quality System [17] was adapted and used as an audit tool (Checklist: Appendix A). The audit tool included four key components for assessment: Organization (Org), personnel (Per), service and satisfaction (Ser), and process improvement (Pro). Each of the components consisted of questions referred to as “Quality System Essentials” with “yes” or “no” responses. These were clear and easy for participants to understand. To determine the percentages, ‘Yes’ responses were coded as 1, ‘No’ responses as 0. The sum totals of ‘Yes’ responses were calculated and reported as percentages for each of the components assessed.

### Data analysis

All data were collected manually on site and then entered onto an excel spreadsheet, cleaned and validated before importing onto Stata for analysis. All statistical analysis was conducted using Stata (version 13). Frequencies and 95% confidence intervals (CI) were estimated for all 11 audited clinics using the t-test. Cohen's d, Hedges' correction and Glass's delta statistical tools were employed to estimate the effect size for the two audit groups, with the Hedges' correction best recommended to minimize bias for sample sizes less than 20 [19, 20]. The Levene’s test defined as an inferential statistic used to evaluate the equality of variances for a variable calculated for two groups or more [21] and an independent samples t-test for two groups, were employed to compare quantitative variables between the pre-test and post-test groups. The relationships between variables were then estimated using the Pearson pair wise correlation coefficient (p) and correlations were reported as significant at p < 0.05 [22].

## Results

Audits were performed at 11 selected rural clinics prior to the introduction of the mLearning intervention. Post-audits were only performed at seven clinics, which were able to access the intervention. In this section the characteristics of the audited rural PHC clinics of KZN are presented, also the pre- and post-audit results, followed by a statistical comparison of the pre- and post-audit results.

### Characteristics of audited rural PHC clinics

Eleven rural PHC clinics from the KZN province in South Africa were pre-audited in March 2021 and post-audited in June 2021. All the audited rural PHC clinics are located more than 10 km away from the nearest town, with nine out of the 11 audited clinics located 10 km or more outside of the nearest hospital. The other two clinics from uMkhanyakude and Ugu districts were shown to have the closest proximity to a hospital (1.1 km and 4.1 km respectively). Due to the gradual phasing out of HIV lay counsellors, HIV testing was found to be performed by a variable number of PHC workers in each of the clinics. Furthermore, four clinics reported to have their HIV testing programmes supported by non-profit organisations on site. Consolidated record keeping of the number of HIV tests performed on a daily basis was therefore reported to be a challenge. None of the audited clinics had access to departmental email addresses and ten clinic managers reported to have been utilising personal emails for communicating with the department of health in the province of KZN. Current status of participating rural PHC clinics’ access to technology resources is illustrated in Table 1. Further participant characteristics are reported in our previous study [9].

**Table 1:** Audited KZN rural PHC clinics access to technology resources

District	Internet provider	Email access	Connectivity status
Amajuba	DOH	Personal	Internet facilities provided, but coverage is poor and internet is slow
Umzinyathi	_	_	No internet access or access to email: clinic relies on the PHC manager at the hospital to deliver printed mail
Umgungundlovu	NGO	Personal	No internet access, email accessed through personal devices
Harry Gwala	DOH	Personal	Wifi provided, however coverage is poor and the internet is very slow, “I was not able to send an email to the clinic until I was able to reach the nearest town.”
Umkhanyakude	_	Personal	No internet facilities provided, the clinic manager uses her personal email on her phone
Zululand	NGO	Personal	No department provided email, but NGO provided for access to emails
KingCetshwayo	NGO	Personal	The router was stolen and not replaced. Email accessed through clinic manager’s personal phone
Ilembe	NGO	Personal	Wifi provided and accessible
Ugu	_	_	The clinic has no internet and no access to email. An attempt to send course access information via whatsapp, however participant could not participate
Ethekwini	DOH	Personal	Wifi connection and smart phones provided, however data gets depleted quickly and top ups are not permissible
Uthukela	_	Personal	The clinic has no access to government provided internet services

### Pre-audit scores for the audited rural PHC clinics in KZN

**Fig. 2:**
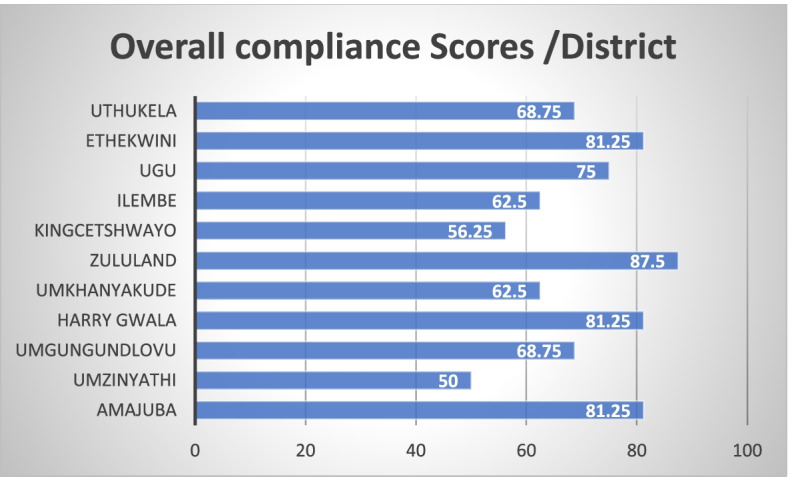
Average quality pre-audit scores per district

Pre-audit results showed that the 11 audited clinics’ average rating scores for compliance to the WHO guidelines for health care facilities in rural and resource-limited settings ranged between poor and moderate quality (50–87.5%). The Umzinyathi (50%) followed by the King-Cetshwayo (56.2%) districts, were rated as the least compliant districts and the Zululand district rated the most compliant (87,5%). Figure 2 illustrates the average pre-audit scores for each of the clinics audited per district.

### Level of compliance to audited WHO HIV rapid testing quality components

**Fig. 3:**
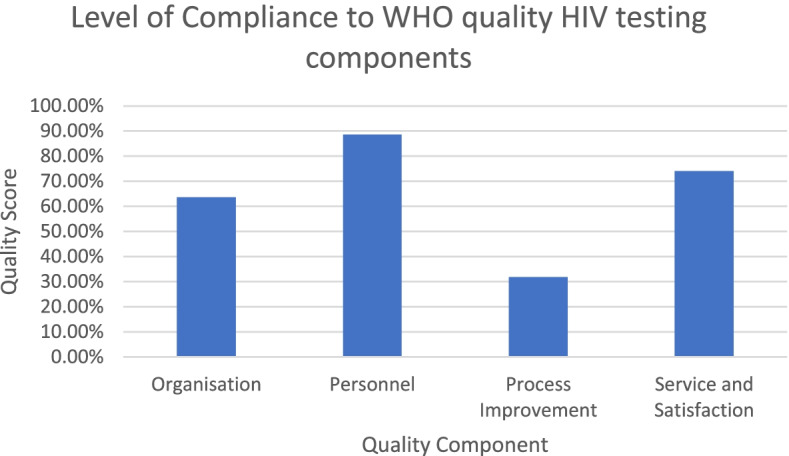
Level of compliance with audited WHO HIV rapid testing quality components

Average responses for each audit component were determined from the sum totals of each of the quality essential questions making up the respective component. For example, the organisation component consisted of three quality essential questions with the following frequencies: 82%, 36% and 73%. Therefore, the consolidated average score for ‘organisation’ was: (82 + 36 + 73)/3 = 61%. Figure 3 illustrates the average responses for each of the audit components: organization, personnel, process improvement and service and satisfaction.

Figure 3 shows that audited clinics scored highest for the personnel component (88%), followed by the service and satisfaction component (74%). They scored moderate for the organisation component (64%) and lowest for the process improvement component (32%).

### Post-audit scores for the audited rural PHC clinics in KZN

**Fig. 4:**
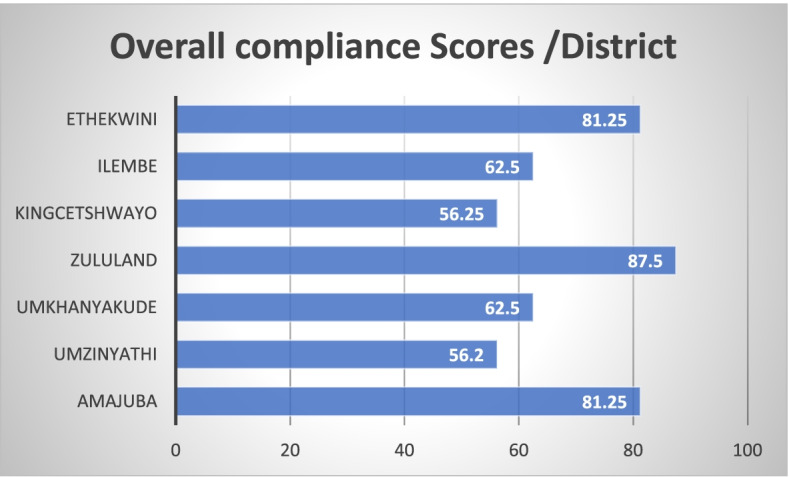
Post-audit average rating scores for each of the clinics audited

The post-audit results showed that the seven audited clinics’ average rating scores for compliance to standard quality guidelines also ranged between poor and moderate quality (56.2—87.5%). The Umzinyathi district (56.3%) rated the least compliant and the Zululand district (87.5) rated the most compliant. The districts that were not able to participate in the mLearning curriculum were the Umgungundlovu, Ilembe, Uthukela and the Ugu districts. Figure 4 illustrates average pre-audit scores for each of the clinics audited per district.

The results obtained at the post-audit were similar to those obtained at the pre-audits for each of the audit components evaluated, with a slight improvement for the Umzinyathi District from 50% to 56,3%. Further statistical results are presented below.

### Statistical tests for differences between pre and post audit results

The t-test and the Levene’s test for independent samples were performed for each of the audit components. Detailed results for the organisation component (Question 1) are presented below, followed by the summary of the results for all the other components in Table 4.

Using an alpha level of 0.05, an independent-samples t-test was conducted to evaluate if the average percentages of tested quality components differed significantly as a function of the pre-test or post-test. For the organization component Q1, the mean (M) for the pre-test was 0.36, and the mean (M) for the post-test was 0.43. The *p*-value was 0.798 and, therefore, the difference between the two means is not statistically significant because the *p*-value is greater than 0.05. In addition, there was an estimated change of 6.5% (SE = 2.49%). This value implies how much the sample mean would vary if the data were to repeat a study using new samples from within a single population. The 95% confidence interval for the average percentage of organization ranged from -0.594 to 0.464. However, there is insufficient evidence (*p* = 0.798) to suggest that both pre and post test results changed the mean for the organization component. In addition, two more measurements from the organization component are reported in Table 2. The results showed that the mean scores for this component revealed a moderate increase from 0.73 to 0.86 in the pre-test, showing an upward growth (Table 2).

**Table 2:** Comparison results for the pre and post audit results for the organization Component Q_1_

Variable		N	Mean	Standard deviation	Standard error mean
Organization Component	Pre-test	11	0.73	0.467	0.141
	Post-test	7	0.86	0.378	0.143

The Levene’s test was also conducted to evaluate whether the population variances for the two groups are equal. The results, *F* = 1.776 and *p* = 0.201, indicate that there is no significant difference between the two variances. We further examined the standard t test, (t = 0.616, *p* = 0.546), which also indicates that there is no significant differences between the pre and post-test groups. The results are illustrated in Table 3.

**Table 3:** Independent samples test

		Levene's Test for Equality of Variances		t-test for Equality of Means				
		F	Sig	t	df	Sig. (2-tailed)	Mean Difference	95% Confidence Interval
Organization Component	Equal variances assumed	1.776	0.201	-0.616	16	0.546	-0.130	(-0.577, 0.317)
	Equal variances not assumed			-0.647	14.891	.527	-0.130	(-0.558, 0.298)

Table 4 illustrates that the Levene’s test and the t-test results for all the quality HIV testing audit components show that there were no significant differences between the pre-test and post-test results, since the *p*-values are greater than 0.05. There was only one exception on one quality essential question: Service and satisfaction (question 3 “When reporting to outside providers, is turnaround time appropriate?”) with *p* = 0.008.

**Table 4:** Stata results for all audited quality essential components

Audit Question	F-test	*p*-value	t-test	*p*-value
Organisation Q_2_	0.222	0.644	-0.260	0.798
Organisation Q_3_	1.766	0.201	-0.616	0.546
Personnel Q_1_	3.073	0.099	-0.789	0.442
Personnel Q_2_	0.222	0.644	-0.260	0.798
Personnel Q_3_	0.415	0.529	0.323	0.751
Personnel Q_4_	0.013	0.912	0.057	0.956
Process impr Q_1_	0.044	0.837	0.459	0.631
Process impr Q_2_	3.073	0.099	-0.789	0.442
Service satis Q_1_	3.073	0.099	-0.789	0.442
Service satis Q_2_	0.222	0.644	0.260	0.798
Service satis Q_3_	9.143	0.008	-1.176	0.257
Service satis Q_4_	0.172	0.684	-0.204	0.841
Service satis Q_5_	0.222	0.644	0.260	0.798
Service satis Q_6_	0.222	0.644	0.260	0.798
Service satis Q_7_	0.222	0.644	0.260	0.798

Similar results were obtained for various questions, most significantly the last three questions under the service and satisfaction component. The similarities in the pre- and post-audit results can be attributed to the validity of the audit results obtained.

### Effect size results

Effect sizes were calculated to determine the practical significance of the study finding, using three techniques; The Cohen’s d, Hedges’ correction and Glass. The Cohen’s d (0.436), Hedges’ correction (0.458) and Glass delta (0.378) results indicated small to medium effect size on overall audit results (0.3 to 0.5), with the Hedges’s correction recommended for small sample sizes also indicating medium effect size. Results for the organisation component (Q1) are illustrated in Table 5 below.

**Table 5:** Independent samples effect sizes

Variable		Standardized	Point estimate	95% CI
Organization Component	Cohen's d	0.436	-.298	(-1.247, 0.660)
	Hedges' correction	0.458	-.284	(-1.187, 0.628)
	Glass's delta	0.378	-.344	(-1.297, 0.636)

## Discussion

Pre- and post-audits were conducted to determine the effect of a mLearning curriculum on quality improvement of HIV rapid testing services in 11 rural clinics of KZN. Audits were conducted on four quality components: organization, personnel, process improvement and service and satisfaction. The overall quality of the HIV testing services was found to have been maintained pre- and post-piloting of the mLearning curriculum, with a slight improvement observed in one of the clinics with the lowest overall compliance scores from 50% to 56,25%. This improvement was a result of a better score obtained for the organisation audit component, which could be attributed to improved access to quality documents. However, the statistics results showed that there were no statistically significant differences between pre- and post-audit results. A significant difference was observed for only one question in the service and satisfaction component; however, this difference was not sufficient to yield a significant effect for the whole component.

Statistical analysis also showed that the differences in pre- and post-audit sample sizes (*n* = 11 and *n* = 7, respectively) had small to medium effect size on the overall audit results. However, since the results showed that there was no statistically significant difference between the two groups, it was not necessary to determine the magnitude of the effect on the overall results [23]. The smaller sample size at post-auditing was due to barriers hindering 36% of the participants from taking full advantage of the proposed intervention. These included poor access to technology devices, and poor and slow internet connections, which led to less participation and to discouragement to complete the course. However, in quasi experimental analysis it is a good indication to establish that the manipulation or intervention did not only influence the mean between observations, but also influenced the standard deviation. Glass et al. (1981) recommends using the standard deviation of the pre-measurement as a standardizer [24]. For the current study, we reported three different techniques for effect size (d), with Hedges’s correction recommended for small sample sizes of less than 12 [23]. Though the differences may have been shown not to be statistically significant, Hedges’s correction indicated medium effect size, which cannot be considered negligible. This indicates that implementing the evaluated intervention in a conducive environment, where participants are afforded enough time and the necessary resources, may yield more positive results as recommended below [23].

To the best of the researcher’s knowledge this was the first study to investigate the effect of a mLearning curriculum for POC diagnostics in resource-limited settings. However, there is available evidence on the evaluation of the effects of mLearning on general nursing education. In contrast to this study, a study evaluating an mLearning module effectiveness to support inter-professional knowledge construction in the health professions, found that the module improved clinical reasoning and inter-professional communication of 98% of participating students [25]. A qualitative Systematic Review study on influences on the implementation of mLearning for medical and nursing education, also found mLearning to be beneficial for good communication and interaction between learners and the learning material for clinical practice [26]. However, similar to the current study, Lall et al. (2019) highlighted concerns over network connectivity and poor device functionality especially in clinical settings, as a barrier to adoption of mLearning. The overall introduction of technology-based interventions such as mLearning has been reported to have a potential to contribute to the provision and access to updated information and training material to facilities in remote areas. Moreover, it has been reported to have had a positive impact on learning motivation and study performance [26, 27]. However, as also observed in the present study, challenging realities for students, doctors, and nurses need to be addressed first [26].

Notable strengths of this study include that more than one test statistic was used to investigate correlations and independent sample effect sizes. This contributed to the reliability of the results obtained. Auditing of the same group which yielded to more coherent results, can also be associated with the reliability and accuracy of the quasi experiment in this study. Limitations to be highlighted in this study include that due to Covid19 restrictions, there were variations in the duration between pre- and post-audits for different clinics. Hence some clinics may have not been sufficiently exposed to the intervention, however the researchers ensured that minimum exposure of 3 months was achieved for all participants. Furthermore, participants were given a platform to express their views as well as evaluate the course for future improvement. Majority of the participants complemented the course content for being relevant and easy to access through Moodle as published in our recent qualitative study [16]. Moreover, 36% of the participating clinics had poor or no internet access and could not participate on the mLearning training and hence were only audited once. However, the conducting of audits of this nature enabled the identification of areas of improvement necessary for future adoption of technology-based interventions.

Based on the identified benefits of using technology-based interventions, as well as the barriers to access and connectivity, this study recommends the provision of small localised WiFi servers located in clinics, which would provide free information to all devices within a limited range. This study also recommends the establishment of internal information sharing platforms at the clinics to improve access to updated quality documents and to encourage interaction between PHC workers. Furthermore, qualitative analysis of the impact of an mLearning curriculum on quality improvement of POC diagnostic services is also recommended to provide more insight on the participants’ experiences.

## Conclusion

The aim of this study to determine the effect of an mLearning curriculum on improving the quality of HIV rapid testing services in rural clinics of KZN, South Africa, was met. The findings of this study showed that the mLearning curriculum had no statistically significant effect on the quality of HIV rapid testing services in participating clinics. However, critical gaps on the lack of provision of reliable technology devices and internet connections were identified and the necessary provisions were recommended to ensure full adoption of technology-based interventions, necessary to improve access to learning material and updated information.

## Supplementary Information

**Additional file 1.**

## Data Availability

Further data leading to and supporting the conclusions of this paper contains confidential information of participating clinics and is stored at the University archives, however it can be made available as supporting documents upon request.

## Abbreviations

AIDS: Acquired Immunodeficiency Syndrome
ART: Anti-retroviral treatment
KZN: KwaZulu Natal
HIV: Human Immunodeficiency Syndrome
eLearning: Electronic Learning
mLearning: Mobile Learning
PHC: Primary Health Care
POC: Point of Care
WHO: World Health Organisation
