# Supplementary material for: The effect of a mobile-learning curriculum on improving compliance to quality management guidelines for HIV rapid testing services in rural primary healthcare clinics, KwaZulu-Natal, South Africa: a quasi-experimental study
Source: BMC Health Serv Res. 2022 May 9;22:624. doi: 10.1186/s12913-022-07978-4 (PMC9081963; doi:10.1186/s12913-022-07978-4)
Supplement: Supplementary file 1 — Additional file 1. [file 12913_2022_7978_MOESM1_ESM.docx]

**Guidelines for Assuring the Accuracy and Reliability of HIV Rapid Testing: Applying a Quality System Approach (WHO, 2005)**

| **Quality System Essential** |  | Yes | No | Assessor’s comments |
| --- | --- | --- | --- | --- |
| **Organization** | Is there a quality policy manual present and accessible? Does the policy manual address all elements of the quality system? |  |  |  |
|  | Does the site have a designated quality ofﬁcer? |  |  |  |
|  | Is the site manager aware of all quality system components? |  |  |  |
| **Personnel** | Does testing staff possess certiﬁcate indicating successful participation in HIV rapid test training? |  |  |  |
|  | Has the staff been oriented to the patient/client ﬂow at the test site? |  |  |  |
|  | Does staff demonstrate professionalism? |  |  |  |
|  | Is number of staff adequate for the site workload?  - Approximately how many tests does each staff member perform per month? |  |  |  |
| **Process Improvement** | •Have any projects been undertaken for process improvement? |  |  |  |
|  | •Has the error rate of POC diagnostics decreased over the past two years? |  |  |  |
| **Service and Satisfaction** | Is staff courteous to clients? |  |  |  |
|  | Are there efforts to reassure client and/ or alleviate client’s fear of needle or sight of blood? |  |  |  |
|  | When reporting to outside providers, is turnaround time appropriate? |  |  |  |
|  | Does the site solicit input and advice from clients? |  |  |  |
|  | Is the testing space adequate in size; clean and well organized? |  |  |  |
|  | Is the environment suitable for patient testing (e.g. temperature, electrical supply)? |  |  |  |
